# Resuscitation of the rare biosphere contributes to pulses of ecosystem activity

**DOI:** 10.3389/fmicb.2015.00024

**Published:** 2015-01-30

**Authors:** Zachary T. Aanderud, Stuart E. Jones, Noah Fierer, Jay T. Lennon

**Affiliations:** ^1^Department of Plant and Wildlife Sciences, Brigham Young UniversityProvo, UT, USA; ^2^Department of Biological Sciences, University of Notre DameSouth Bend, IN, USA; ^3^Department of Ecology and Evolutionary Biology and CIRES, University of ColoradoBoulder, CO, USA; ^4^Cooperative Institute for Research in Environmental Sciences, University of ColoradoBoulder, CO, USA; ^5^Department of Biology, Indiana UniversityBloomington, IN, USA

**Keywords:** CO_2_ pulses, dormancy, desiccation, dominance, stable isotope probing (SIP), soil rewetting, seed bank, rarity

## Abstract

Dormancy is a life history trait that may have important implications for linking microbial communities to the functioning of natural and managed ecosystems. Rapid changes in environmental cues may resuscitate dormant bacteria and create pulses of ecosystem activity. In this study, we used heavy-water (H^18^_2_O) stable isotope probing (SIP) to identify fast-growing bacteria that were associated with pulses of trace gasses (CO_2_, CH_4_, and N_2_O) from different ecosystems [agricultural site, grassland, deciduous forest, and coniferous forest (CF)] following a soil-rewetting event. Irrespective of ecosystem type, a large fraction (69–74%) of the bacteria that responded to rewetting were below detection limits in the dry soils. Based on the recovery of sequences, in just a few days, hundreds of rare taxa increased in abundance and in some cases became dominant members of the rewetted communities, especially bacteria belonging to the Sphingomonadaceae, Comamonadaceae, and Oxalobacteraceae. Resuscitation led to dynamic shifts in the rank abundance of taxa that caused previously rare bacteria to comprise nearly 60% of the sequences that were recovered in rewetted communities. This rapid turnover of the bacterial community corresponded with a 5–20-fold increase in the net production of CO_2_ and up to a 150% reduction in the net production of CH_4_ from rewetted soils. Results from our study demonstrate that the rare biosphere may account for a large and dynamic fraction of a community that is important for the maintenance of bacterial biodiversity. Moreover, our findings suggest that the resuscitation of rare taxa from seed banks contribute to ecosystem functioning.

## Introduction

In nature, most microorganisms live in unpredictable environments and experience conditions that are suboptimal for growth and reproduction. Some organisms attempt to maximize their long-term fitness by dispersing offspring into new and hopefully better habitats. Other organisms hedge their bets by entering a reversible state of reduced metabolic activity in a process known as dormancy. Dormancy builds seed banks, which are reservoirs of inactive individuals that can potentially be resuscitated in the future under a different set of environmental conditions (Lennon and Jones, 2011). Dormancy can protect taxa from extinction by buffering against demographic and environmental stochasticity (Kalisz and McPeek, 1992; Honnay et al., 2008). It can also reduce the strength of species interactions and allow taxa to coexist via the storage effect (Chesson and Warner, 1981). Recently, efforts have been made to integrate dormancy into ecosystem models by accounting for the physiological processes and energetic requirements associated with the active and inactive members of a microbial community (Stolpovsky et al., 2011; Wang et al., 2014a,b). These studies suggest that, in addition to being an important diversity-maintaining mechanism, dormancy may have important implications for understanding and predicting ecosystem processes.

Soil microorganisms play an essential role in regulating critical ecosystem processes, such as carbon sequestration, nutrient cycling, and the flux of trace gasses. Growing evidence suggests, however, that only a small fraction of the bacterial community may be responsible for soil processes occurring at any given point in time. In some cases, it is estimated that >90% of the microbial biomass is inactive, 50% of all bacterial taxa are dormant, and at least 25% of all soil genomes contain genes that enable individuals to be resuscitated from a dormant state (Alvarez et al., 1998; Lennon and Jones, 2011; Wang et al., 2014a). In particular, microbial activity can be extremely low in dry soils owing to a combination of desiccation stress and the reduced diffusion of substrates (Schimel et al., 2007). Under these relatively inactive conditions, precipitation events serve as an environmental cue that terminates microbial dormancy in dry soils (Saetre and Stark, 2005; Placella et al., 2012). As soils are rewetted, there is an increase in microbial metabolism (Iovieno and Baath, 2008; Blazewicz et al., 2013) that corresponds with pulses of ecosytem activity. Within hours of a precipitation event, CO_2_ production can be 500% higher than pre-wetting conditions (Fierer and Schimel, 2003) and when scaled over longer time periods, moisture-mediated pulses of ecosystem activity contribute up to 25% of the carbon budget in some terrestrial ecosystems (Schimel et al., 2007). Furthermore, recent studies suggest that historical exposure to soil moisture regimes may select for bacteria with functional traits that confer tolerance to drying and rewetting events (Evans and Wallenstein, 2014). Taken together, drying and rewetting events offer an ideal situation to evaluate the interactions between dormancy, microbial diversity, and ecosystem processes.

Resuscitation of dormant microbes may also provide an opportunity to explore the functional importance of the “rare biosphere.” The rare biosphere is a term that was coined to describe the observation that most microbial taxa are extremely uncommon (Sogin et al., 2006). If microbial taxa contribute to ecosystem processes in proportion to their abundance then it may not be critical to focus on the rare biosphere (see Grime, 1998). However, it is well established that some rare groups of bacteria contribute disproportionately to certain biogeochemical processes. For example, a specific subset of rare methane oxidizing bacteria regulated methane emissions from riparian floodplains (Bodelier et al., 2013), while sulfate reduction in a peatland ecosystem was attributed to a single genus of bacteria that comprised less than 0.006% of the total microbial community (Pester et al., 2010). It is also important to consider that the relative abundance of bacterial populations can be highly variable through time due to fluctuations in environmental conditions (Pedrós-Alió, 2012; Hugoni et al., 2013; Shade et al., 2014). For instance, over half of the bacterial taxa in the Chesapeake Bay cycled between being abundant and rare over a 3 year period (Campbell et al., 2011). Previous work has suggested that shifts in the commonness and rareness of bacterial taxa may be due to transitions between active and inactive metabolic states (Jones and Lennon, 2010), but few studies have linked these dormancy dynamics to environmental change and pulses of ecosystem activity.

In this study, we explore the effects of soil moisture variability on bacterial resuscitation and ecosystem processes. After documenting pulses of trace gasses (CO_2_, CH_4_, and N_2_O) in both field experiments and laboratory microcosms, we used heavy-water stable isotope probing (SIP) to identify bacteria that were resuscitated from a state of low metabolic activity based on the incorporation of ^18^O into their DNA following the rewetting of dry soils. We demonstrate that a large number of rare taxa rapidly responded to shifts in soils moisture and contributed to pulses of ecosystem activity. Our findings suggest that shifts in environmental cues can affect the dormancy of bacterial communities in ways that maintain biodiversity and influence ecosystem processes.

## Materials and methods

### Study site

Our study took place at the W. K. Kellogg Biological Station (KBS) Long Term Ecological Research (LTER) site in southwestern Michigan, USA. We conducted field and laboratory experiments using land-use treatments that simulate some of the major ecosystem types found in the Upper Great Lakes region of North America, specifically agricultural crop rotation (T1), successional grassland (T7), deciduous forest (DF), and coniferous forest (CF) (Robertson et al., 2000). Average annual precipitation at the KBS LTER is 890 mm (±148.0 SD, *n* = 21) with half falling as snow, and the mean annual temperature is 9.0°C (±0.81 SD, *n* = 21, http://lter.kbs.msu.edu). All soils are fine-loamy, mixed, mesic Typic Hapludalfs with an average pH of 6.0 and a cation exchange capacity of approximately 5.5 cmol kg^−1^.

### Pulses of ecosystem activity: field experiment

Prior to pursuing more mechanistic experiments, we conducted a field experiment to assess the water-limitation of microbial processes in our relatively mesic habitat. Over a 17-day period (June 15–July 2 2007), we manipulated rainfall by evenly dispensing 5 mm of distilled water onto a 3 × 3 m plot in one of the replicate agricultural field sites (T1) on days 4, 7, and 14 of the experiment. Before initiating the experiment, we deployed environmental sensors at 2 cm depth to quantify the temporal dynamics of soil CO_2_, soil moisture, and soil temperature. The placement of the sensor near the soil surface allowed us to capture an integrated CO_2_ response to moisture before the gas was released to the atmosphere (Riveros-Iregui et al., 2007; Aanderud et al., 2011). We measured CO_2_ concentrations (ppmv) using non-dispersive infrared absorption with a 3% CO_2_ GMT222 sensors (Vaisala, Helsinki, Finland), while monitoring soil moisture (m^3^ H_2_O m^−3^ soil) and temperature (°C) with ECH2O-TE sensors (Decagon Devices, Pullman WA, USA). Data from the sensors were generated every 10 s, averaged on a 30 min time-interval, and stored on field data loggers (CR1000, Campbell Scientific, Inc., Logan UT, USA). We analyzed the resulting data on a 12-h time-step using a time series multiple regression model:

(1) $$CO_{2}(t)=CO_{2}(t-1)+moisture(t)+moisture(t-1)+\varepsilon_{t}$$

where *t* is the current time step, *t* – 1 is the previous time step, and ε_*t*_ is the residual error. We corrected for non-random distributions of the residuals using methods described elsewhere (Aanderud et al., 2011, 2013).

### Pulses of ecosystem activity: microcosm experiment

To gain insight into the microbiological contributions to pulsed ecosystem activity observed in the field, we performed a more controlled rewetting experiment in the laboratory. The microcosm approach was also used for the stable isotope probing (SIP) experiments, which we describe in the next section. Following the summer dry-down of the soils in July 2008, we sampled soils from three of the replicated plots from the four ecosystem types (agricultural crop rotation, successional grasslands, deciduous forests, and CFs). We removed 10 soil cores (0–5 cm soil depth) from randomly selected locations in each the three replicate plots with a soil corer (5 cm length × 2 cm width) and homogenized the soils to create 12 composite samples (4 ecosystem types × 3 replicates). The soils were immediately brought back to the laboratory and passed through a 2 mm sieve. In triplicate, we dispensed 3 g of field-dry soil (≈0.05 g H_2_O g soil^−1^) into 40 mL borosilicate glass vials with septated screw caps. For each ecosystem type, we randomly assigned three microcosms to a dry treatment (no water added). The remaining microcosms belonged to the rewetting treatment and received 0.6 mL of H_2_O. We then incubated all 24 microcosms for 96 h at 25°C in a temperature-controlled incubator. During the experiment, we collected 1 mL of gas from the headspace of each microcosm every 12 h. With these gas samples, we quantified CO_2_ using a LI-820 infrared gas analyzer (Lennon et al., 2012). In addition, we measured CH_4_ and N_2_O using gas chromatography [Hewlett Packard 5890 Series II, Rolling Meadows, IL, USA, Ruan and Robertson (2013)]. We calculated the net production of trace gasses (μg C-CO_2_, C-CH_4_, or N-N_2_O g soil^−1^) by summing the amount of gas generated during each of the eight 12-h increments and tested for the effect of rewetting on the gas production using Two-Way ANOVA and Tukey's HSD tests.

### Stable isotope probing (SIP)

Using the microcosm approach described above, we identified bacteria that were resuscitated by rewetting using H^18^_2_O-DNA SIP. We initiated SIP by adding 0.6 mL of H^18^_2_O (97 atom% ^18^O; Isotech, Sigma-Aldrich, St. Louis, MO, USA) to a dry soil sample and incubating it for 72 h at 25°C. This rewetting created a five-fold increase in gravimetric moisture for all soil samples (dry soil ≈0.05 g H_2_O g soil^−1^, rewetted soil ≈0.25 g H_2_O g soil^−1^). As a control, we also used SIP to characterize the bacterial composition of the dry soils. This was done by adding 0.6 mL of H^18^_2_O to a soil sample and immediately stopping bacterial activity by transferring the microcosm to −80°C. We then followed the ultracentrifugation, gradient fractionation, and DNA recovery procedures of SIP described in detail elsewhere (Schwartz, 2007; Aanderud and Lennon, 2011). Briefly, at least 1 μg of genomic DNA was extracted from soils using a PowerSoil DNA Isolation Kit (MoBio, Carlsbad, CA, USA), and was loaded into 4.7 mL OptiSeal polyallomer tubes (#361621, Beckman Coulter Inc., Brea, CA, USA) containing cesium trifluoroacetate (CsTFA, #17-0847-02, GE Healthcare, Salt Lake City, UT, USA) with a buoyant density 1.61 g mL^−1^ (Leigh et al., 2007). Each 4.7 mL tube received approximately 2.9 mL of CsTFA and 1.75 mL of nuclease-free H_2_O. The tubes were placed into a TLA 110 rotor and spun at 178,000 rcf (64,000 rpm) for 48 h at 20°C. After centrifugation, we collected 20 fractions (235 μL each) from each tube using a digitally controlled fractionator. We identified the unlabeled bacterial DNA in the dry treatment and ^18^O-labeled bacterial DNA in the rewetted treatment by performing qPCR on all gradient fractions via amplification of the 16S rRNA gene (Aanderud and Lennon, 2011). All of the above was done for a total of 24 samples (4 ecosystems × 2 watering treatments [dry vs. rewetted] × 3 replicates).

### Bacterial community responses to rewetting

We characterized the bacterial communities in the dry and rewetted soils from the SIP samples using bar-coded sequencing of the 16S rRNA gene. We PCR-amplified the V1–V2 hypervariable region of the 16S rDNA gene using the bacterial primers 27F and 338R with unique 12-nt error correcting Golay barcodes (Fierer et al., 2008). The thermal cycle conditions were as follows: an initial denaturation step at 94°C for 3 min followed by 35 cycles of denaturation at 94°C for 45 s, annealing at 50°C for 30 s, and an extension at 72°C for 90 s. After pooling PCR amplicons at approximately equimolar concentrations, samples were sequenced at the Environmental Genomics Core Facility at the University of South Carolina in a 454 Life Sciences genome sequence FLX (Roche, Branford, CT, USA) instrument. All sequences were analyzed using mothur (v.1.29.2) an open-source, expandable software pipeline for microbial community analysis (Schloss et al., 2009). After removing barcodes and primers, we eliminated sequences that were <250 bp in length and sequences that had homopolymers longer than 8 bp. In addition, we denoised the sequences with AmpliconNoise (Quince et al., 2011). Finally, we removed chimeras using UCHIME (Edgar et al., 2011), along with chloroplast, mitochondria, archaeal, eukaryotic, and unknown rRNA gene sequences according to the Ribosomal Database Project (Cole et al., 2009). We then aligned our sequences against the SILVA database (Pruesse et al., 2007) with the SEED aligner, created operational taxonomic units (OTUs) based on uncorrected pairwise distances at the 97% sequence similarity level, and determined the phylogenetic identity of OTUs using the SILVA database.

To assess the effects of soil rewetting on bacterial communities from different ecosystems, first, we used multi-level partial least squares discriminant analysis (PLS-DA) and permutational multivariate analyses of variance (PERMANOVA). PLS-DA is an ordination technique that is especially suited to deal with datasets where there are a larger number of predictors (e.g., OTUs) than observations (samples), while alleviating problems arising from multicollinearity (Barker and Rayens, 2003; Pérez-Enciso and Tenenhaus, 2003). Importantly, PLS-DA allowed us to accommodate the paired nature of our experimental design (i.e., the non-independence between a dry and rewetted sample). PLS-DA was implemented with the mixOmics package in R (Dejean et al., 2013). While the PLS-DA aided in the visualization of our data, we tested for the main effects and interaction between the rewetting treatment and ecosystem type using PERMANOVA (Anderson, 2001), which was performed with the *adonis* function in the vegan package in R (Oksanen et al., 2013). Second, we quantified the compositional turnover that occurred for each experimental unit between the dry and rewetted time points using the Bray-Curtis dissimilarity index. Last, we quantified bacterial richness in our samples as the observed number of OTUs after rarefaction. The effects of rewetting and ecosystem type on richness were evaluated using Two-Way ANOVA with Tukey's HSD tests.

### Resuscitation of rare bacteria

Unlike other properties of biological diversity (i.e., richness and evenness), there are few widely accepted ways to quantify rarity (Gaston, 1994). Often, somewhat arbitrary cutoffs are used (e.g., <0.1% of total recovery) to determine whether or not a taxon is considered rare. In this study, we made inferences about the putative contributions of rare bacteria to ecosystem activity by characterizing shifts in the rank abundance of taxa in response to rewetting. First, we determined the number of OTUs that were present in both dry and rewetted soils (i.e., “shared”), along with the number of OTUs that were present in either the dry soils or rewetted soils (i.e., “unshared”). With this information, we defined a rare responder as a taxon that was below our detection limits in the dry sample, but recovered in the same experimental unit after rewetting. In addition to visualizing changes in relative recovery of OTUs with rank abundance curves, we tested for differences in the recovery of rare bacteria in the four ecosystems using One-Way ANOVA and Tukey's HSD tests. Last, taxonomic trends of rare responders in some of the major phyla and classes were shown in a heat map with hierarchal clustering using the *heatmap* function in the gplot package in R (Warnes et al., 2014).

## Results

### Pulses of ecosystem activity

**Figure 1:**
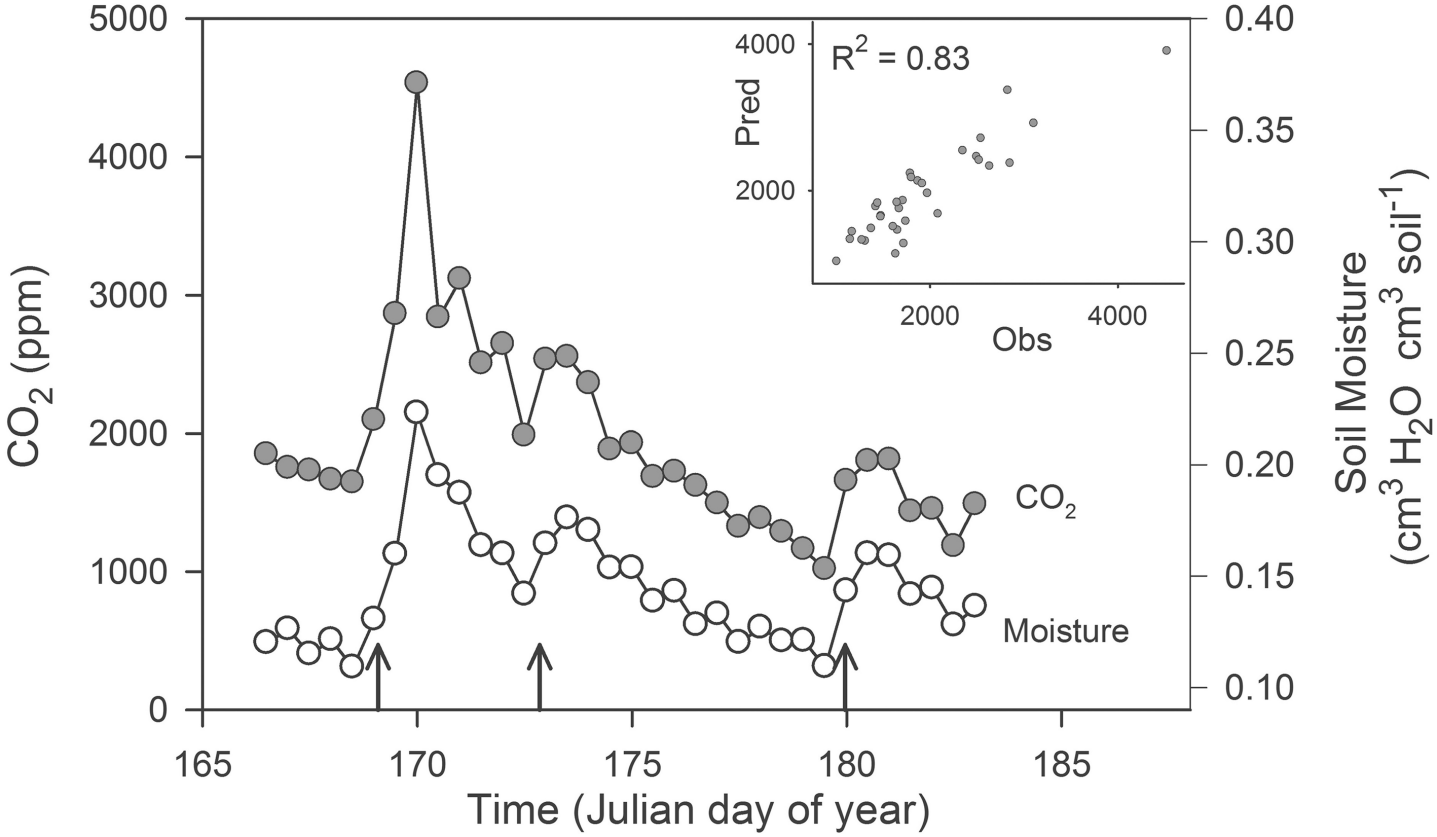
**Pulses of ecosystem activity in an agricultural ecosystem stimulated by experimental rain events (upward pointing arrows)**. The inset panel is a plot of the observed (Obs) and predicted (Pred) CO_2_ generated from a multiple regression model. We estimated soil CO_2_ concentrations (2 cm depth) and soil moisture (0–5 cm) using real-time sensor data averaged on a 12 h time-step from 15 June 2007 (day 166) through 3 July 2007 (day 184).

Microbial communities responded rapidly to soil rewetting and this resuscitation corresponded with pulses of ecosystem activity. In our field experiment, soil moisture increased in the agricultural site by at least 2.5-fold following each of the three simulated rainfalls. These rewetting events generated pulses of CO_2_ that lasted more than 2 days (Figure 1). The autoregressive soil moisture model explained the majority of the observed variation in soil CO_2_ concentrations (*R*^2^ = 0.83). We found that *CO_2_(t)* was positively correlated with *moisture(t)* (23,292 ± 18.9 [mean ± SE], ppmv CO_2_/cm^3^ H_2_O cm^−3^ soil, *t*_5.3_ = 1232, *P* < 0.0001), but was negatively correlated with *moisture(t – 1*) (−3949 ± 25.7 [mean ± SE], ppmv CO_2_/cm^3^ H_2_O cm^−3^ soil, *t*_5.3_ = −153.7, *P* < 0.0001).

**Figure 2:**
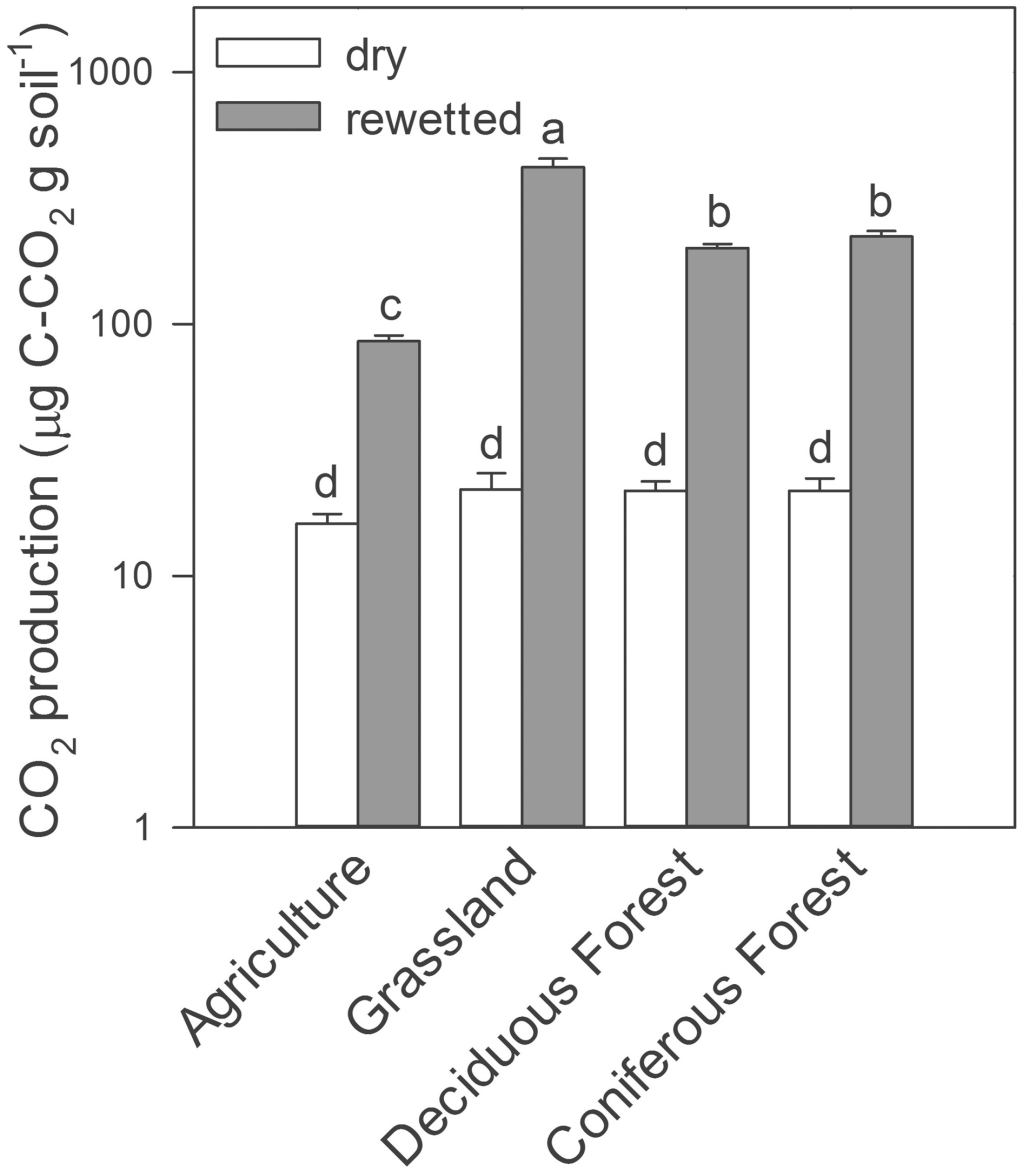
**CO_2_ production from laboratory microcosms with dry and rewetted soils obtained from four ecosystem types**. Values are means ± SEM (*n* = 3) with letters indicating differences (*P* < 0.05) based on a Two-Way ANOVA and Tukey's HSD test.

Similarly, rewetting altered trace gas production in the soil microcosm experiments conducted in the laboratory. Rewetting increased gravimetric soil moisture by a factor of five (≈0.05–0.25 g H_2_O g soil^−1^). As a result, we observed up to a 20-fold increase in CO_2_ production in rewetted soils compared to dry soils, irrespective of ecosystem type (Two-Way ANOVA, ecosystems × water treatment, *df* = 3, *F* = 202, *P* = 0.001, Figure 2). With the exception of soils from the agricultural site, CH_4_ production was lower in rewetted soils than dry soils (Two-Way ANOVA, ecosystems × water treatment, *df* = 3, *F* = 26.6, *P* = 0.002, Supplemental Figure 1). Last, rewetting increased N_2_O production in grassland soils but decreased production in deciduous forest soils (Two-Way ANOVA ecosystems × water treatment, *df* = 3, *F* = 10.3, *P* = 0.022).

### Stable isotope probing (SIP)

SIP was effective at distinguishing bacteria that resuscitated following soil rewetting. Based on the qPCR results, the dry treatment contained unlabeled DNA in fractions 12 and 13 with a buoyant density in CsTFA ranging from 1.531 to 1.548 g mL^−1^, and the rewetted treatment contained ^18^O-labeled DNA in fractions 9 and 10 with buoyant density in CsTFA ranging from 1.574 to 1.585 g mL^−1^ (Supplemental Figure 2). Thus, rewetting led to a 0.026–0.054 g mL^−1^ increase in the buoyant density of ^18^O-labeled bacterial DNA. We used the DNA in fractions 12 and 13 to represent the bacterial communities in dry soil conditions and the DNA in fractions 9 and 10 to represent the bacterial communities in the rewetted soils.

### Bacterial community responses to soil rewetting

**Figure 3:**
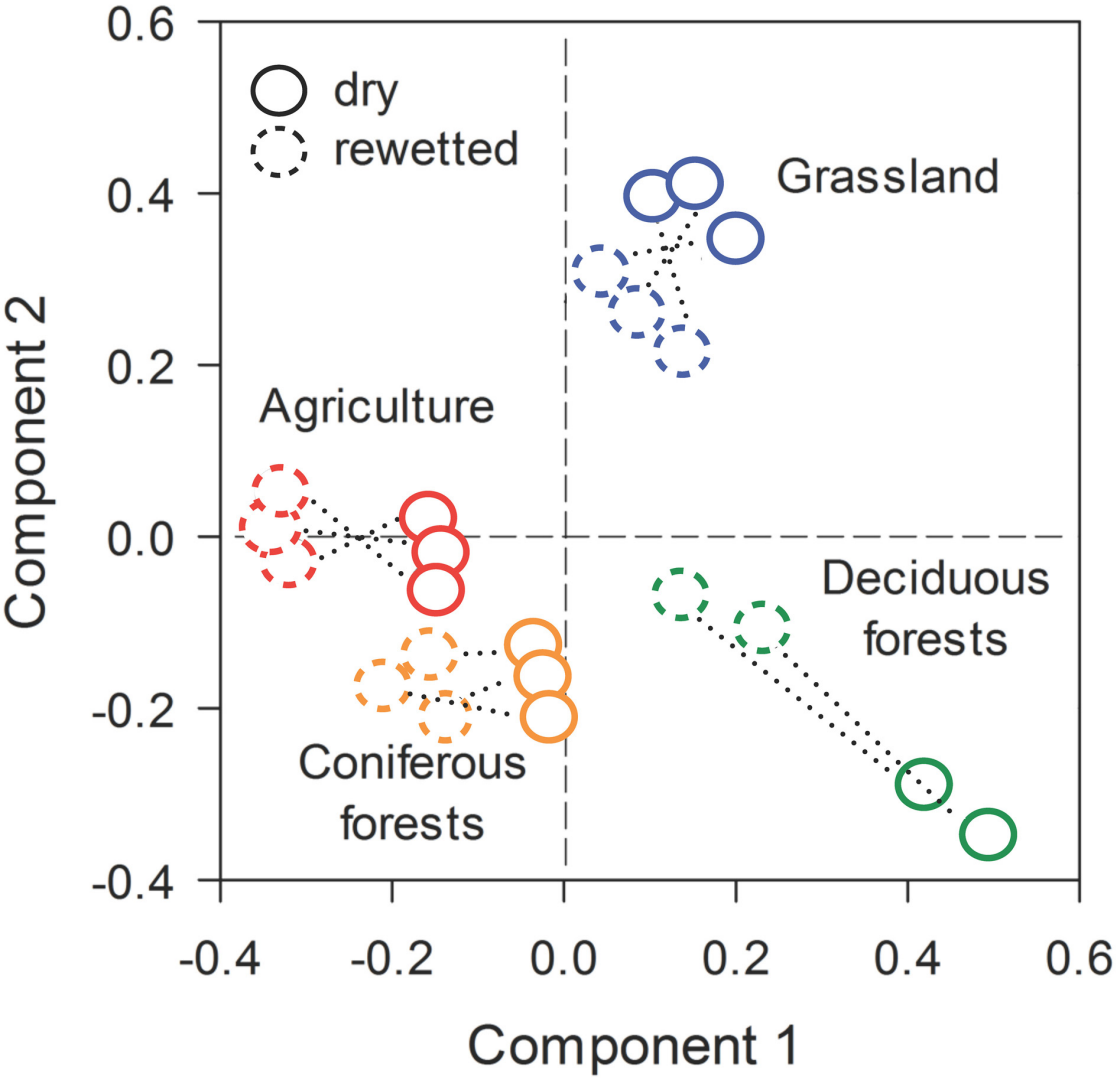
**Stable isotope probing (SIP) revealed rapid shifts in bacterial community composition in soils that were rewetted with heavy water (H^18^_2_O)**. The multivariate ordination was generated using Partial Least Squares Discriminant Analysis (PLS-DA) on a sample × OTU matrix that took into account ecosystem type and pairs of dry-rewetted samples (indicated by dashed lines).

Across all ecosystems, rewetting had strong effects on bacterial community composition. This inference was based on the recovery of 29,931 quality sequences and 9256 unique OTUs (BioProject ID: PRJNA269181, http://www.ncbi.nlm.nih.gov/bioproject/). One of the 24 samples (a replicate from the deciduous forest) was not included in our analyses due to a large proportion of low quality sequences. Prior to rewetting, PLS-DA results demonstrated that bacterial communities from the dry soils separated in ordination space based on ecosystem type. After rewetting, bacterial communities retained a signature of the ecosystem from which they were derived, but were separated in ordination space relative to the dry conditions (Figure 3). PERMANOVA results supported these interpretations: both ecosystem type (*P* = 0.008) and rewetting (*P* = 0.002) had a strong effect on bacterial composition. There was a marginally significant ecosystem × rewetting interaction on composition (*P* = 0.08), suggesting that deciduous forest communities may have been more responsive than bacterial communities from the other ecosystems (see Figure 3).

The significant effects of rewetting were associated with rapid turnover in bacterial composition. Based on Bray-Curtis pairwise comparisons, bacterial composition diverged by 65–74% during the rewetting period. Turnover was slightly higher in the deciduous forest and grassland sites than soil bacteria from the CF (One-Way ANOVA, *df* = 7, *F* = 5.20, *P* = 0.03, Figure 3). Despite large and rapid shifts in composition, rewetting did not affect bacterial richness within an ecosystem (Supplemental Figure 3).

### Resuscitation of rare bacteria

**Figure 4:**
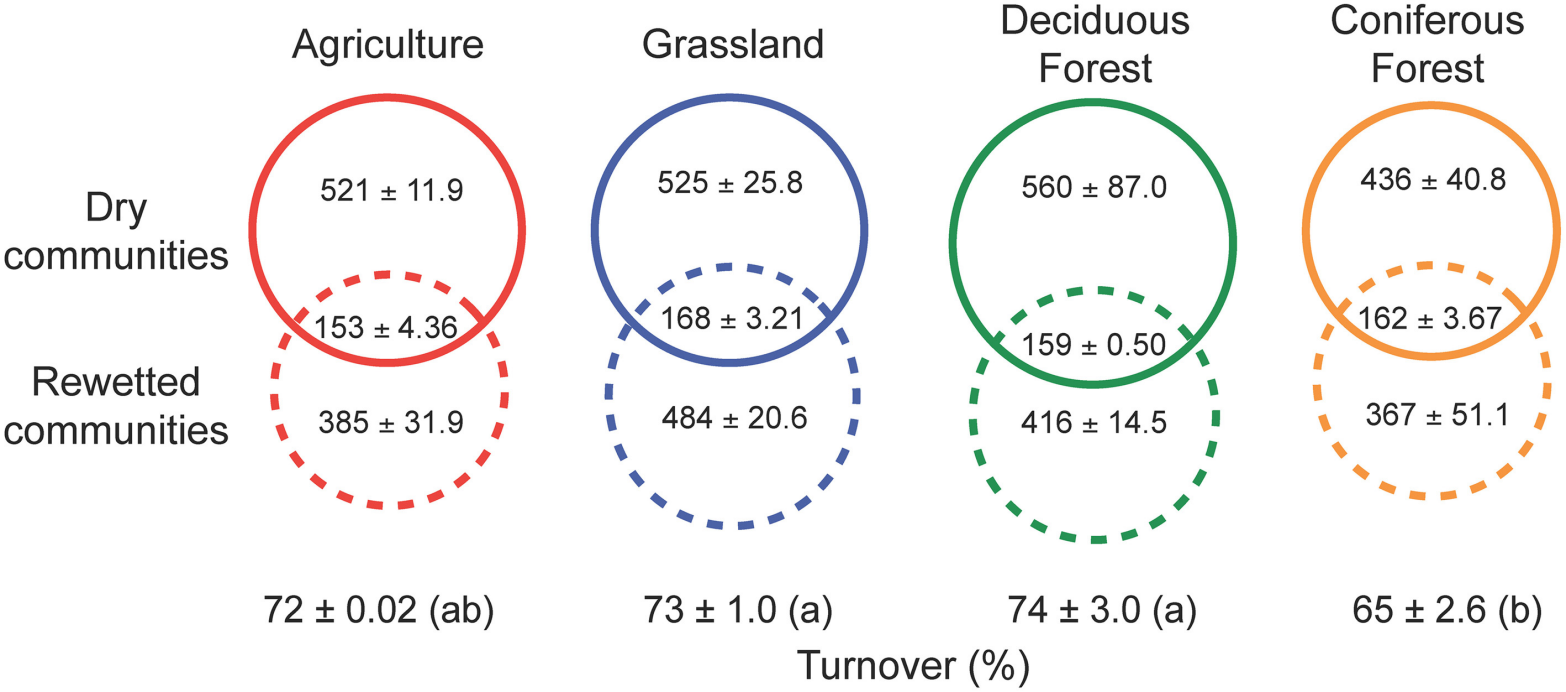
**Resuscitation of rare bacteria contributed to shifts in bacterial composition following the rewetting of soils with heavy water (H^18^_2_O)**. High rates of turnover (estimated using Bray-Curtis dissimilarity) were due in large part to the recovery of rare OTUs that were below detection limits in the sampling of dry soils. The numbers of rare OTUs (means ± SEM) are presented in dashed solid circles, while the shared OTUs occurring in both dry and rewetted soils are present in the intersection of the dashed and solid circles. OTU numbers are based on the observed number of OTUs present after rarefaction by sequence number. Different letters indicate significant differences (*P* < 0.05) based on a One-Way ANOVA and Tukey's HSD test.

Our results indicate that the rewetting of dry soil resuscitated rare bacterial taxa. Irrespective of ecosystem type, a large fraction (69–74%) of the OTUs recovered after rewetting was not detected from the paired sample under dry conditions (Figure 4). Some of the taxa that responded to rewetting were also recovered in the initial, dry samples (26–31%). Within this shared pool, 45–55% of the OTUs were comprised of singletons and doubletons in dry soils, lending further support to the view that pulses of ecosystem activity were associated with the resuscitation of rare taxa.

**Figure 5:**
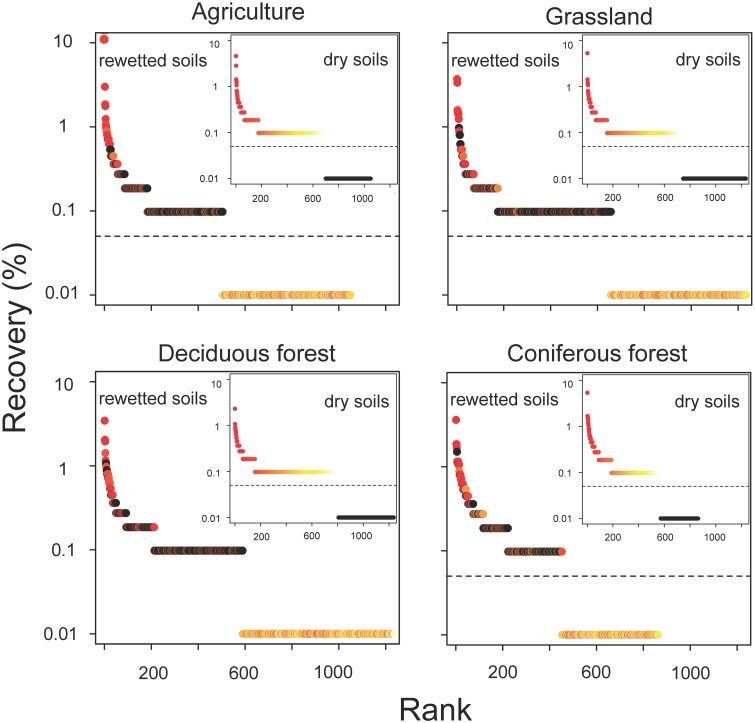
**Resuscitation resulted in dynamic shifts in the rank abundance of rare bacteria in rewetted soils**. OTUs are color-coded based on their ranked recovery in the dry soils (inset panel): red taxa had the highest recovery, yellow the lowest, and black were below detection in the initial (dry) sample. OTUs with ranks falling below the dashed horizontal line were below detection limits.

**Figure 6:**
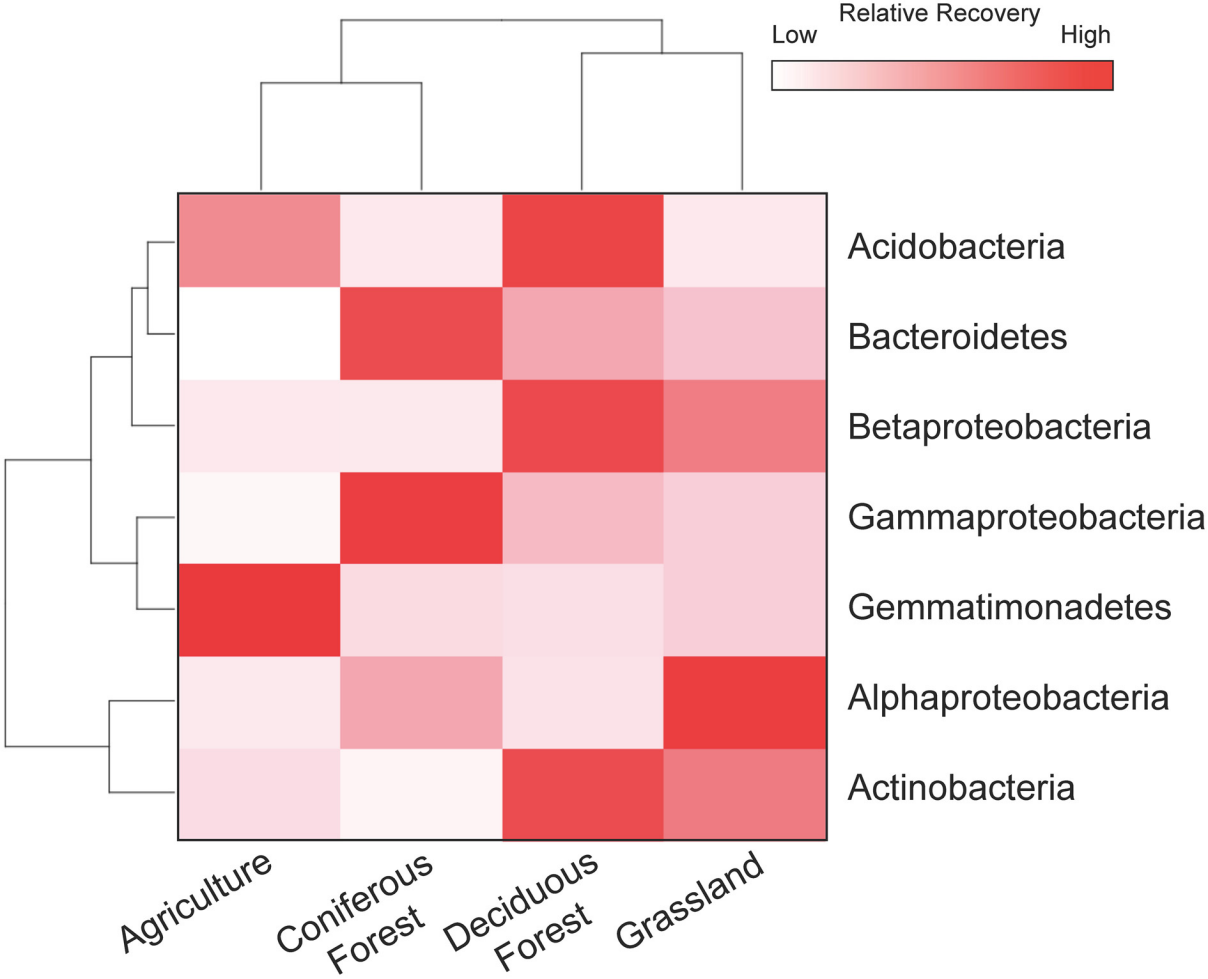
**Rare bacteria demonstrated ecosystem-specific responses to rewetting**. Heat map showing the distribution of rare OTUs for four phyla and three Proteobacteria subclasses that contributed ≥ 1% to the total recovery of rewetted communities. Values are based on means with hierarchal clustering of ecosystem (bottom) and phylum (left).

The resuscitation of rare taxa suggests that the dominance structure of soil bacterial communities may be highly dynamic. This view is supported by large shifts in the rank abundance distributions of rare taxa across ecosystem types (Figure 5). In each ecosystem, hundreds of rare taxa, which were below detection limits in the dry soils, increased in recovery and rank after rewetting. Together, these rare OTUs comprised 48–59% of the sequences that were recovered in the rewetted samples. The contribution of rare OTUs in the rewetted samples varied among ecosystems. There was higher recovery of rare responders in grassland and deciduous forests than agriculture sites and CF communities (One-Way ANOVA, *df* = 3, *F* = 7.66, *P* = 0.01). Across all ecosystems, 13 rare OTUs became dominant members (≥1% recovery) of rewetted communities and were repeatedly ranked in the top 11 taxa of the different communities. Of these rare responders, most (85%) were Proteobacteria, with 38% belonging to the Alphaproteobacteria family Sphingomonadaceae; 23% belonging to the Betaproteobacteria family Comamonadaceae; and 15% belonging to the Betaproteobacteria family Oxalobacteraceae. Despite larger responses of the aforementioned taxa, rare bacteria were recovered in all of the major phyla and classes found in our samples. However, the response of these coarse taxonomic groups to rewetting was ecosystem-specific. The recovery of rare Alphaproteobacteria, Betaproteobacteria, Gammaproteobacteria, and Gemmatimonadetes varied among the four ecosystems (Figure 6). For example, the recovery of rare Betaproteobacteria was at least two-fold higher in grasslands and deciduous forests than the two other ecosystems and Gemmatimonadetes were 2.2-times higher in agricultural sites than the three other ecosystems.

## Discussion

Results from our stable isotope probing (SIP) allowed us to identify a diverse array of fast-growing soil bacteria that were associated with pulses of ecosystem activity. A large fraction of these bacteria (69–74%) consisted of rare taxa, which accounted for 60% of the 16S rRNA reads in rewetted soil samples. Our findings suggest that rare taxa are important for the maintenance of soil bacterial diversity and that the resuscitation of these taxa from seed banks contributes significantly to soil processes like CO_2_, CH_4_, and N_2_O production.

### Rewetting and pulses of ecosystem activity

Using field manipulations and laboratory microcosm experiments, we observed that dry soils generated large pulses of ecosystem activity when they were rewetted. The CO_2_ pulses in our field study were ephemeral and closely tracked soil moisture dynamics during drying and rewetting (Figure 1), while microcosm experiments revealed that CO_2_, CH_4_, and N_2_O production were strongly influenced by rewetting (Supplemental Figure 1). Moisture-mediated pulses of ecosystem activity have been observed in a variety of habitats and are a well-recognized phenomenon in soil science (Birch, 1964; Fierer and Schimel, 2003; Lee et al., 2004; Jenerette et al., 2008). Rewetting is thought to stimulate microbial activity via two primary mechanisms. First, increases in soil moisture release microorganisms from desiccation stress (Lennon et al., 2012). Second, microorganisms encounter high concentrations of resources during rewetting events. Although rewetting may enhance the availability of substrates within the soil matrix and make protected soil organic matter more accessible, many studies suggest that resuscitated microorganisms are consuming the cellular constituents of other microorganisms (Fierer and Schimel, 2003; Xiang et al., 2008). For example, it is well documented from work with isolates that some microorganisms produce and accumulate osmolytes as an adaptive response to desiccation stress (Csonka, 1989). In nature where soil moisture is dynamic, compatible solutes (e.g., proline, glycine, betaine) need to be disposed of to maintain osmotic equilibrium. It has been argued that the release of microbial osmolytes during rewetting may be responsible, at least in part, for pulses of ecosystem activity observed terrestrial ecosystems (Schimel et al., 2007, but see Boot et al., 2013). Regardless of the exact mechanisms, it is well estalished that at least some microorganisms undergo rapid transitions from low to high activity when dry soils are rewetted, and that these changes in metabolism have consequences for ecosystem proceses.

### Rare bacteria contributed to pulses of ecosystem activity

Our results revealed that rare bacterial taxa contributed to pulses of ecosystem activity following rewetting. In this study, we conservatively classified rare taxa based on the detection limits of our sequencing. Specifically, if a taxon was recovered in a rewetted sample, but not in the paired dry sample then we considered it rare. Based on this logic, we found that rare bacteria comprised 69–74% of taxa and nearly 60% of the 16S rRNA gene sequences in rewetted communities, irrespective of the ecosystem sampled. Many of the sequences recovered from our soil samples likely came from heterotrophic microorganisms. We assume that when these bacteria became labeled with ^18^O they generated CO_2_ as a byproduct of both anabolic and catabolic processes. A much smaller fraction of the bacteria that responded to rewetting were recovered in the initial dry sample (26–31%). Of these shared taxa, approximately 50% were represented in the dry sample by either singletons or doubletons, which lends further support to the view that pulses of ecosystem activity were associated with the resuscitation of rare taxa.

Over the past decade, there has been considerable interest in the “rare biosphere” (Sogin et al., 2006; Hugoni et al., 2013; Logares et al., 2014). Most scientists agree that rare taxa are important for cataloging biodiversity, but it is less clear whether or not they are important for ecosystem processes. For example, the core microbiome refers to a collection of organisms that are consistently encountered among similar habitats, and therefore is thought to be essential for carrying out vital processes (Shade and Handelsman, 2012). Rare taxa have a lower probability of being considered part of the core microbiome, and as a result, it is hypothesized that these taxa may contribute minimally to ecosystem processes (Pedrós-Alió, 2012). For example, a taxon may be rare if by chance it disperses into a local community from a large pool of regional species (Pedrós-Alió, 2006). In this case, an organism may find itself in an environment for which it is not particularly well adapted. It is predicted that these transient microbes will have low rates of metabolism, and thus contribute minimally to ecosystem functioning (Pedrós-Alió, 2012). There are also “resident” rare taxa, which may have different ecological strategies and metabolic profiles. Some rare taxa may be consistently active, but have very slow growth rates (Hugoni et al., 2013). Other groups of taxa may be conditionally rare with the potential to rapidly respond to environmental change through shifts in physiology (Shade et al., 2014), including resuscitation from dormancy (Lennon and Jones, 2011).

Our findings are consistent with the view that rare species perform essential functions in an ecosystem. It has long been recognized that the removal of some rare taxa can have a large effect on ecosystem processes (Paine, 1966). For example, in a recent meta-analysis of macroscopic organisms (i.e., coral reef fishes, alpine plants, and tropical trees), it was shown that functional trait diversity could largely be attributed to rare species (Mouillot et al., 2013). In microbial systems, the direct manipulation of rare taxa via dilution has been shown to affect soil processes, including the establishment of pathogens (van Elsas et al., 2012) and rates of nitrogen cycling (Philippot et al., 2013).

### The identity of rare responders

Based on the design of our SIP experiment, the bacteria responding to rewetting can be viewed as fast-growing taxa. Many of these bacteria were initially rare but became dominant members of the community following rewetting. Taxa belonging to the Sphingomonadaceae (Alphaproteobacteria) were one such group of bacteria that responded to rewetting. Many representatives of the Sphingomonadaceae are aerobic, heterotrophs (Reddy and Garcia-Pichel, 2007; Kyselková et al., 2009) that exhibit extreme metabolic versatility as evidenced by their ability to use organic substrates ranging from glucose to aromatic hydrocarbons (Alonso-Gutiérrez et al., 2009; Xie et al., 2011; Regonne et al., 2013). Previous studies in a Mediterranean grassland also documented that the sphingomonads are responsive to soil rewetting events (Placella et al., 2012). In addition, some members of the Betaproteobacteria responded to rewetting. For example, the Comamonadaceae and Oxalobacteraceae are root- and rhizosphere-associated bacteria (Ofek et al., 2012; Dibbern et al., 2014) that are generally recognized as fast-growing organisms. Together, representatives of these families accounted for 38% of the ^18^O-lableed taxa in our study. Last, our results suggest that a few fast-growing taxa were potentially stimulated by microbial byproducts generated during soil rewetting (i.e., methane). For example, as methane declined in rewetted soils (Supplementary Figure 1), we observed an increase in the recovery of taxa belonging to the Methylocystaceae (Alphaproteobacteria), which are known methanotrophs (Gulledge et al., 2001).

Several phyla and classes of bacteria exhibited ecosystem-specific responses to soil rewetting. For example, the recovery of rare Gemmatimonadetes was higher in agricultural sites than the other ecosystems investigated in this study. Gemmatimonadetes are abundant in ecosystems that experience low levels of moisture and frequent soil drying (DeBruyn et al., 2011). The lack of irrigation in our agricultural sites combined with the high rates of evapotranspiration from the fields may have increased the relative recovery of these taxa. Also, the recovery of rare Betaproteobacteria was higher in grasslands and deciduous forests. These two ecosystems support diverse plant communities and high levels of primary productivity and Betaproteobacteria was possibly stimulated by the flush of photosynthate or the variety of root exudates accompanying rewetting (Fierer et al., 2007; Eilers et al., 2010).

Recent studies have demonstrated that H^18^_2_ O-DNA SIP can be an effective tool for linking microbial taxa to ecosystem processes that are influenced by moisture availability (Aanderud and Lennon, 2011; Adair and Schwartz, 2011; Woods et al., 2011). We assume that while bacteria were growing and incorporating ^18^O into their DNA, they were also contributing to the pulses of ecosystem activity that resulted from the rewetting. However, there are a number of important caveats that should be highlighted. First, some bacteria may have responded to rewetting but used H^18^_2_O to meet catabolic maintenance energy demands to sustain existing cells (van Bodegom, 2007) or for the upregulation of cellular machinery for growth, such as RNA, ribosomes, and amino acids (Blazewicz et al., 2013). We would not expect to recover these taxa in our DNA-based analyses. Second, other soil organisms (e.g., archaea, fungi, and nematodes) could have contributed to the observed pulses of ecosystem activity, but our PCR primers did not capture the response of these taxa to rewetting. Last, not all bacteria that were recovered with our sequencing contributed to the pulses of ecosystem activity that we measured. For example, chemolithoautotrophic bacteria do not produce CO_2_ as a byproduct of their metabolism. Therefore, our H^18^_2_O DNA-SIP captured some of the rare bacteria that *did not* contribute to processes, but also missed other microorganisms that *did* contribute to processes.

### Dynamic rank abundance distributions

In addition to linking rare bacteria to pulses of ecosystem activity, our results provide insight into how these taxa might contribute to the maintenance of biodiversity. All else being equal, rare species have a higher probability of going extinct (locally or globally) than common species (Lawton et al., 1994). However, there are some advantages to being rare, such as reduced risk of predation and parasitism, especially for asexually reproducing organisms like most bacteria (Pedrós-Alió, 2006). Previous work has shown that common bacteria were comprised largely of dormant individuals, while rare taxa were disproportionately more active (Jones and Lennon, 2010). Although based on a snapshot in time, these findings suggest that transitions into and out of dormancy could lead to dynamic rank abundance distributions (Lennon and Jones, 2011). The rare biosphere most likely contains both dormant taxa, as well as active but slow-growing taxa. As such, it is important to emphasize that the rare biosphere is not synonymous with a seed bank. Being rare does not necessarily imply dormancy, just as being abundant does not necessarily imply high metabolic rates. Our results revealed that many (but not all; see Figure 4) rare taxa with relatively low levels of metabolic activity were capable of responding to an environmental cue (e.g., moisture). This view is consistent with recent findings, which report that many habitats (e.g., air, skin, oceans, gut, etc.) are comprised of conditionally rare taxa (Shade et al., 2014). In other words, stochastic or predictable changes in the environment may cause large changes in the relative abundance of microbial taxa in space or time.

The results from the current study provide support for the notion of a dynamic microbial rank distribution. Dry soils were comprised of bacteria that on an aggregate level had low levels of metabolic activity. We observed a high degree of compositional turnover (65–74%) in just a few days. This pattern could be attributed to the resuscitation of rare OTUs that were below detection in the largely dormant dry soil. By this definition, approximately 60% of all sequences from the rewetted soils could be attributed to rare taxa. Hundreds of bacterial taxa were not only metabolically resuscitated, but also reproduced fast enough to become dominant members of the community (Figure 5). However, the temporal resolution of our data only allows us to speculate about the persistent effects of moisture-mediated resuscitation on bacterial community composition. For example, it is possible that rewetting only created ephemeral “blooms” of fast growing bacteria. Tracking taxa through repeated drying and rewetting cycles would provide a test of whether or not resuscitated bacteria retain a high rank or if they fall back into the tail of the rank abundance distribution. In addition, future studies could evaluate the importance of deterministic vs. stochastic processes that influence bacterial responses to rewetting. Our findings suggest that bacteria within an ecosystem type responded similarly to changes in soil moisture (Figure 3), but it is unclear whether or not there is long-term coherence in the relative abundance of microbial taxa following environmental change. In sum, our study suggests that rare bacteria may not be just transient members of the community; at least in some cases, these taxa are recruited into dominant roles due to environmental fluctuations as they exit dormancy (see Shade et al., 2014).

## Conclusion

Shifts in environmental cues can affect the dormancy of microbial communities, specifically member of the rare biosphere, in ways that maintain biodiversity and influence ecosystem processes. Rewetting stimulated the growth of rare bacteria, which increased their rank abundance, and contributed to ecosystem processes disproportionately to their recovery in dry soils. Thus, rewetting provides evidence that rapid changes in environmental conditions may cause dynamic shifts in rank abundance among bacteria and helps maintain the high levels of biodiversity in soils. Owing to contributions of rare species to essential ecosystem processes, more attention needs to be directed toward understanding microbial seed banks and the functional importance of the rare biosphere.

## Author contributions

Zachary Aanderud and Jay Lennon designed the study. Zachary Aanderud, Jay Lennon and Noah Fierer conducted the experiments. Zachary Aanderud, Jay Lennon, Noah Fierer, and Stuart Jones analyzed and interpreted the data, helped write and review the manuscript. Both Zachary Aanderud and Jay Lennon agree to be accountable for all aspects of the work in ensuring that questions related to the accuracy or integrity of any part of the work are appropriately investigated and resolved.

### Conflict of interest statement

The authors declare that the research was conducted in the absence of any commercial or financial relationships that could be construed as a potential conflict of interest.
